# Comparison of Vascular Perturbations in an A**β**-Injected Animal Model and in AD Brain

**DOI:** 10.4061/2011/918280

**Published:** 2011-09-29

**Authors:** Nattinee Jantaratnotai, Jae K. Ryu, Claudia Schwab, Patrick L. McGeer, James G. McLarnon

**Affiliations:** ^1^Department of Anesthesiology, Pharmacology and Therapeutics, Faculty of Medicine, The University of British Columbia, 2176 Health Sciences Mall, Vancouver, BC, V6T 1Z3, Canada; ^2^Department of Pharmacology, Faculty of Science, Mahidol University, Rama VI Road, Phayathai, Bangkok 10400, Thailand; ^3^Kinsmen Laboratory of Neurological Research, Department of Psychiatry, Faculty of Medicine, The University of British Columbia, Vancouver, BC, V6T 1Z3, Canada

## Abstract

The validity of amyloid-*β* peptide (A*β*_1-42_) intrahippocampal injection, as an animal model of Alzheimer's disease (AD), has previously been considered in terms of inflammatory reactivity and neuronal damage. In this work, we have extended the testing of the animal model to vasculature by comparison of selected properties of microvessels *in vivo* with those in human AD brain tissue. The injection of A*β*_1-42_, relative to control PBS (phosphate buffered saline), increased the mean number of microvessels and diminished the mean length of microvessels in the molecular layer of dentate gyrus. The animal model showed A*β*_1-42_, but not PBS, injection was associated with abnormalities in morphology of microvessels which were characterized as looping, fragmented, knob-like, uneven, and constricted. In particular, numbers of constricted microvessels, defined as vessels with diameters less than 3 *μ*m, were considerably enhanced for A*β*_1-42_, compared to PBS, injection. In comparison, human AD brain demonstrated an elevated number of microvessels with a diminished mean length relative to nondemented (ND) brain. Additionally, microvessel perturbations in AD brain showed a similar pattern of morphological abnormalities to those observed in A*β*_1-42_-injected rat hippocampus. Constricted microvessels were a prominent feature of AD brain but were rarely observed in ND tissue. These results provide the first evidence that a peptide-injection animal model exhibits a commonality in perturbations of microvessels compared with those evident in AD brain.

## 1. Introduction

 A host of animal models have been proposed with relevance to modeling the pathological features which characterize Alzheimer's disease (AD) brain. In order to test the validity of the animal models, properties and predictions from animal models can be compared with the characteristic features present in AD brains. These features involve a broad spectrum of altered properties from those of control nondemented (ND) individuals including the presence of enhanced deposits of amyloid-*β* peptide (A*β*) [1], neurofibrillary tangles (NFTs) [2], abnormalities in vasculature [3–5], evidence for ongoing chronic inflammation [6, 7], and loss of neurons and synaptic connectivity [8]. Advancement, fine tuning, or rejection of animal models involves a rigorous and complex comparative process concerning the testing of many variables. 

 Intrahippocampal injection of A*β*_1−42_ in rat brain has been suggested as an animal model which emphasizes the inflammatory reactivity present in human AD brain [9]. This model shows marked enhancement of microgliosis in response to peptide relative to control injections of PBS (phosphate buffered saline) vehicle or reverse peptide (A*β*_42-1_). In addition, hippocampal neuronal loss is significantly increased with A*β*_1−42_, compared to control, injection. Importantly, drug inhibition of microglial inflammatory responses has a demonstrated efficacy for conferring neuroprotection [10–12]. Comparison at the molecular and cellular levels has included the finding that enhanced expression of the purinergic ionotropic P2X7 receptor in activated microglia occurred similarly both in the animal model and in AD brain tissue [13].

 Another feature of the A*β*_1−42_ rat model is evidence for an inflammatory response involving altered vasculature including a leaky blood-brain barrier (BBB). In this case A*β*_1−42_ induced an increased permeability of BBB compared to control PBS injection allowing infiltration of plasma protein into parenchymal brain regions [12]. Elevated brain fibrinogen was suggested as an amplifying factor for microglial activation and inflammatory reactivity. Overall, the results from *in vivo* studies indicated that A*β*_1−42_ injection elicited microglial reactivity which may be associated with a weakened BBB. These findings enhanced creditability of the model for predicting vascular changes since evidence has suggested BBB in AD brain may be damaged [14].

 The overall purpose of the present study was to test and extend the utility of A*β* peptide intrahippocampal injection as an animal model of AD. The present work was specifically designed to compare vascular perturbations in the animal model with specific microvessel changes and irregularities evident between AD and ND brain tissue. The comparison has included numbers and lengths of microvessels and abnormalities in microvessels including vessels with constricted diameters.

## 2. Materials and Methods

### 2.1. Animal Model Study

 All protocols involved studies on male Sprague-Dawley rats and were approved by the University of British Columbia Animal Care Ethics Committee. The relevance of using intrahippocampal A*β*_1−42_ as an animal model of AD has been reviewed [9], and the overall procedures employed in use of the model have been detailed in previous work from this laboratory [10–13]. Briefly, stereotaxic injection of peptide (2 nmol of full length A*β*_1−42_; California Peptide, Napa, CA) or control (PBS) was performed into the dentate gyrus region of hippocampus (AP: −3.3 mm, ML: −1.6 mm, DV: −3.2 mm). Injection of peptide, compared with PBS, precipitates an enhanced microglial inflammatory response accompanied by substantial loss of hippocampal neurons.

### 2.2. Tissue Preparation and Immunohistochemistry

 At one week postinjection brains were removed and postfixed and cryoprotected prior to cutting into 40 *μ*m sections. Free-floating sections were processed for immunohistochemistry with sections incubated overnight at 4°C with primary antibodies to rat endothelial cell antigen (RECA-1, 1 : 1000; Serotec, Oxford, UK) or laminin (1 : 1000; Sigma, St. Louis, MO). The use of RECA-1 and laminin for staining of microvessels has been detailed in [12]. Sections were incubated in Alexa Fluor conjugated secondary antibodies (1 : 200; Invitrogen, Carlsbad, CA) for immunofluorescence staining. In immunostaining controls, primary antibody was omitted in all staining procedures. The tissues were examined under a Zeiss axioplan-2 fluorescent microscope (Zeiss, Jena, Germany) using a DVC camera (Diagnostic Instruments, Sterling Heights, MI) with Northern Eclipse software (Empix Imaging, Mississauga, ON, Canada). Quantitative assessment was performed under a constant predefined light setting. The digitized images were analyzed using NIH ImageJ 1.37 b software (National Institute of Health, Bethesda, MD). Four coronal hippocampal sections (200 *μ*m apart) were used for quantitative analysis (detailed procedures in [10–13]). In each stained section, four nonoverlapping fields within the granule cell layer and molecular layer regions were selected (magnification of 40x). Density and lengths of microvessels were measured in molecular layer regions of dentate gyrus; mean values for these variables were found from averaged values over 1.3 mm^2^ areas. In some experiments numbers of microvessels with narrow diameters were measured. These microvessels are defined by diameters ≤3 *μ*m and termed constricted.

### 2.3. Human Brain Tissue

 Postmortem medial temporal cortical (MTC) tissue was obtained from the Kinsmen Laboratory brain bank at the University of British Columbia (Vancouver, BC, Canada). Protocols for use of human tissue were in accordance with ethical guidelines established by The University of British Columbia. Brain tissue from MTC was obtained from 9 ND cases (75–99 years of age; mean 83.0 ± 2.5) and 9 AD cases (65–90 years of age; mean 76.2 ± 2.8). Neuropathological criteria were used in the classification of brain tissue with ND cases showing no clinical or pathological history of dementia or other neurological disorders. AD cases were characterized by immunohistochemical assessment of the density of plaques and NFT in limbic and neocortical areas (including hippocampus, amygdala, and frontal, temporal, parietal, and occipital cortex). All AD cases conformed to criteria provided by the National Institute on Aging and Reagan Institute [15].

### 2.4. Immunohistochemistry

 The protocols used for laminin immunohistochemical staining in AD and ND brain tissue have been detailed [12, 16]. Briefly, free-floating sections with 40 *μ*m thickness were cut from medial temporal cortical tissue. Sections were then transferred into PBS/Triton-X solution and incubated overnight at room temperature with laminin (1 : 1000, Sigma). Biotinylated secondary antibody was applied followed by incubation in avidin-biotinylated horseradish peroxidase complex with labeling visualized by incubation in diaminobenzidine (DAB) solutions. Sections were then washed and mounted on glass slides, air-dried, and coverslipped; immunostaining controls were performed using standard procedures omitting primary antibodies; under these conditions no control staining was observed for any marker. The images were acquired using a light microscope (Olympus BX51) and a digital DP71 camera (Olympus, Center Valley, PA). Detailed quantification method has been described previously [16]. Four sections in the grey matter of medial temporal cortex were used for analysis with fields separated by fixed distances to ensure no overlap between image areas. Laminin staining was used to measure numbers and lengths of microvessels in 1 mm^2^ cortical areas. Constricted microvessels, with diameters ≤3 *μ*m, were also measured in the cortical brain regions. Quantitative assessment was performed under a constant predefined light setting. The digitized images were analyzed using NIH ImageJ 1.37 b software (National Institute of Health, Bethesda, MD). 

### 2.5. Statistical Analysis

 Values are expressed as means ± SEM. Statistical significance was assessed by one-way ANOVA, followed by Student-Newman-Keuls multiple comparison test (GraphPad Prism 3.0). Significance was set at *P* < 0.05.

## 3. Results

### 3.1. Microvessels in the A*β*_1−42_ Intrahippocampal Injection Animal Model

#### 3.1.1. Numbers and Lengths of Microvessels

**Figure 1:**
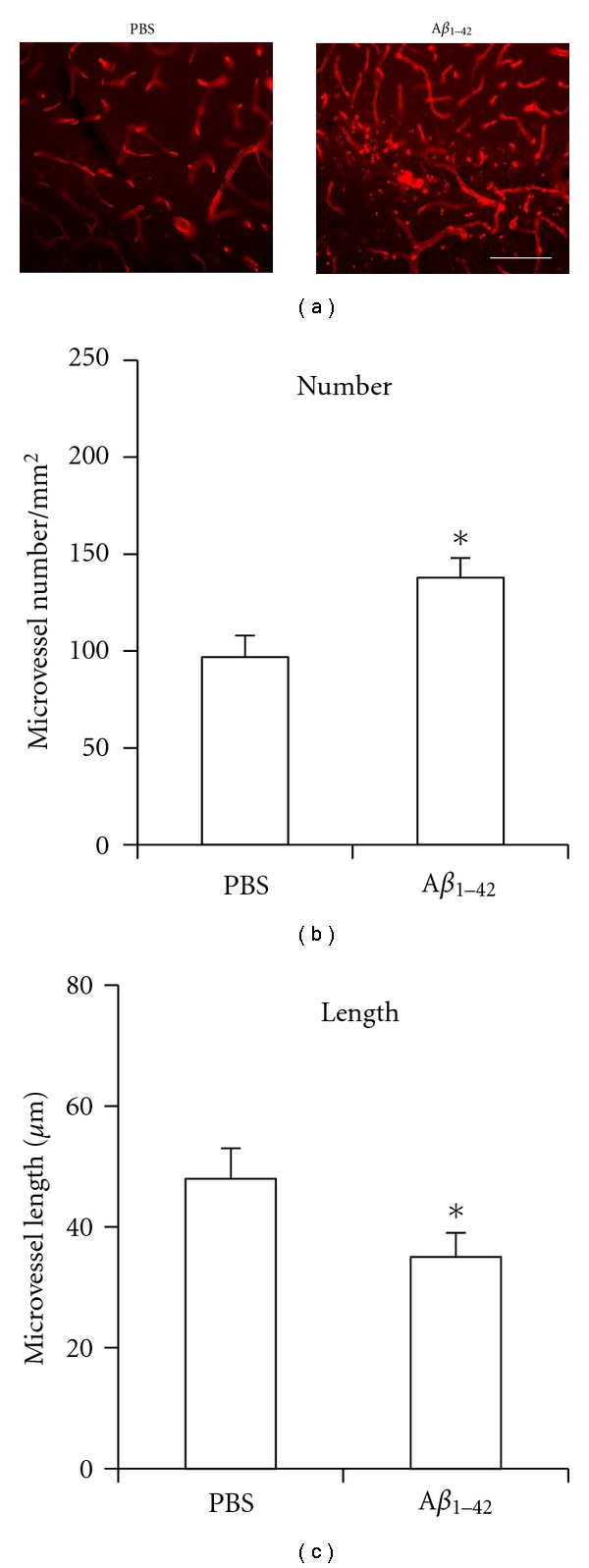
Representative patterns of RECA-1 staining in rat hippocampus. (a) Microvessels in control (PBS) injected rat hippocampus (left panel) and microvessels in A*β*_1−42_-injected hippocampus (right panel). Scale bar is for 70 *μ*m. (b) Bar graph for the number of microvessels/mm^2^ (*n* = 5 each). (c) Bar graph for microvessel length (*n* = 5 each). *P ≤ 0.05 for A*β*_1−42_ versus PBS.

Typical patterns of microvessel staining (RECA-1 marker) are shown for control PBS vehicle (Figure 1(a), left panel) and A*β*_1−42_ (Figure 1(a), right panel) at 7 d after injection in rat hippocampus. The area of staining was for the molecular layer of dentate gyrus. Although morphological differences were observed between the two animal groups (see below), the most evident difference was the increased number of microvessels in peptide-injected brain. The numbers of microvessels/mm^2^ for control and A*β*_1−42_ are presented in the bar graph of Figure 1(b). Also shown are the mean lengths of microvessels for the two animal groups (Figure 1(c)). Overall (*n* = 5 each for control and A*β*_1−42_ group), peptide-injected brain demonstrated a 42 ± 7% increase in numbers of microvessels, normalized to area, compared to control PBS injection. Mean length of microvessels was significantly decreased (by 27 ± 4%) in A*β*_1−42_, compared with PBS, injection. Although the increase in microvessel density could indicate angiogenesis, it should be noted that a specific angiogenic marker would be required to assess formation of new blood capillaries in peptide-injected brain.

#### 3.1.2. Morphology of Microvessels in A*β*_1−42_-Injected Rat Hippocampus

**Figure 2:**
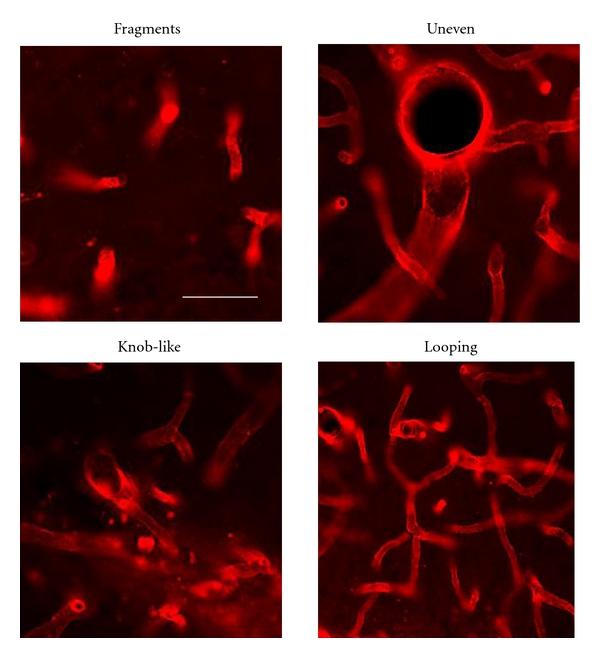
Morphology of microvessels stained with RECA-1 in A*β*_1−42_-injected hippocampus. Panels show morphological features including fragments, looping microvessels, and vessels with knob-like and uneven diameters. The scale bar represents 40 *μ*m.

 Inspection of the morphology of microcapillaries indicated enhanced irregularities in vessels from peptide-injected hippocampus compared with control (Figure 2). For example, fragmented microvessels were much more common after A*β*_1−42_ compared with PBS, injection. Other microvessel abnormalities which were relatively abundant following peptide injection included constricted microcapillaries (see below) and microvessels with looping, knob-like, and uneven appearances (Figure 2). Although these morphological categories largely reflect subjective descriptions, such characteristics were not commonly observed in PBS-injected rat hippocampus. 

#### 3.1.3. Constricted Microvessels in A*β*_1−42_-Injected Rat Hippocampus

**Figure 3:**
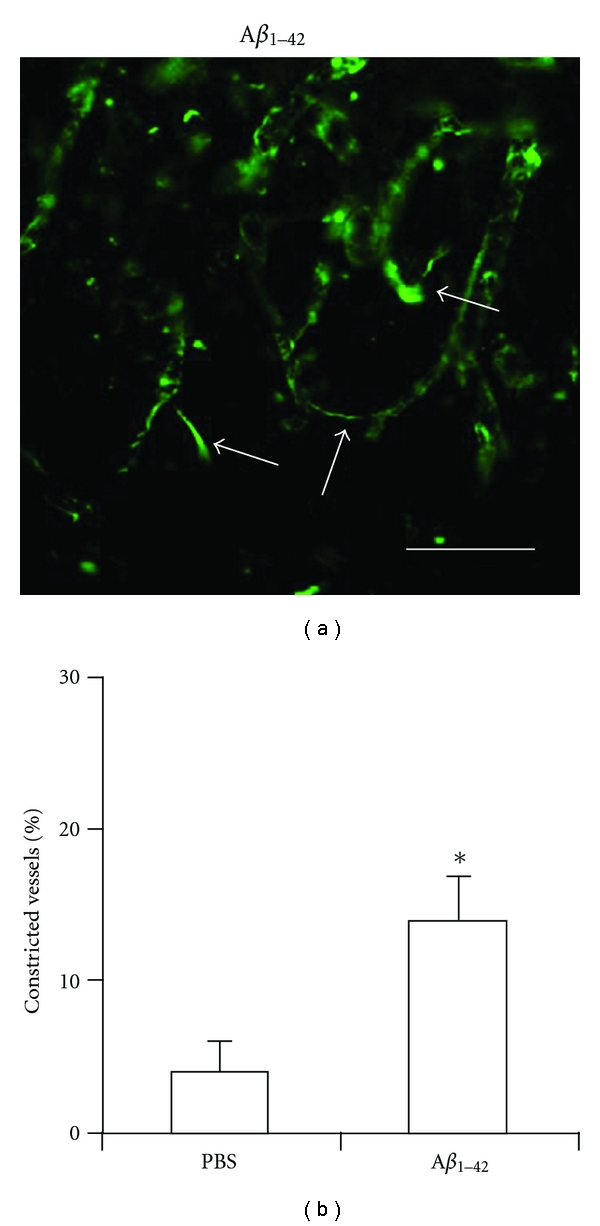
Constricted microvessels in A*β*_1−42_-injected hippocampus. (a) Laminin immunoreactivity showing examples of constricted microvessels (indicated by arrows). The scale bar is for 50 *μ*m. (b) Bar graph showing % of constricted microvessels for PBS control and A*β*_1−42_ injection (*n* = 5). *P ≤ 0.05 for A*β*_1−42_ versus PBS.

 A general finding in peptide-injected hippocampus was the appearance of constricted microvessels. These narrow microcapillaries (laminin staining, Figure 3(a)) exhibited 3 *μ*m or less in diameters and often showed attachment to larger width microvessels. In some cases the constricted microcapillaries formed a bridge between adjacent larger blood vessels. Overall (*n* = 5, Figure 3(b)), 14 ± 3.1% of microvessels in A*β*_1−42_-injected rat hippocampus demonstrated constricted diameters; the corresponding value in PBS-injected brain was 4 ± 1.4%. At present, it is not known if the constricted microvessels represent angiogenic vessels or possibly a population of damaged vessels.

### 3.2. Microvessels in AD and ND Brain Tissue

#### 3.2.1. Numbers and Lengths of Microvessels

**Figure 4:**
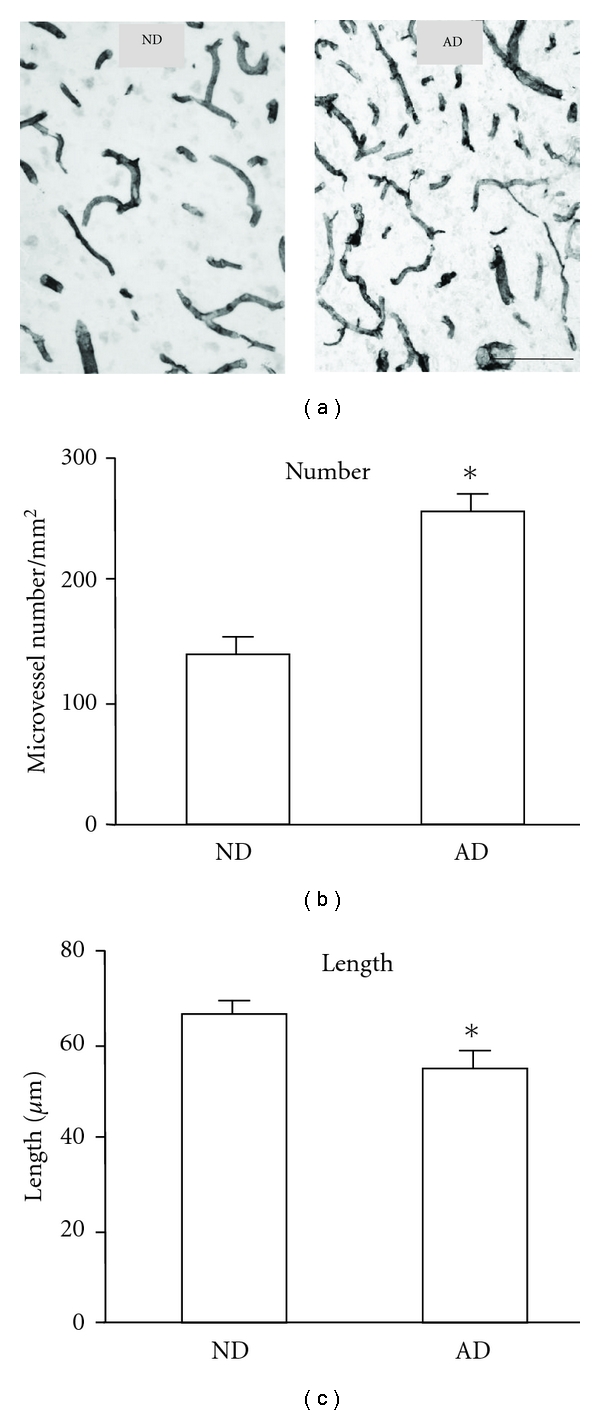
Representative laminin staining for microvessels in human ND and AD brain tissue. (a) Microvessels in ND (left panel) and AD (right panel). The scale bar represents 100 *μ*m. (b) Bar graph for mean numbers of microvessels/mm^2^. (c) Bar graph for microvessel length (*μ*m) (ND, *n* = 9; AD; *n* = 9); *P ≤ 0.05 for AD versus ND.

 Remodeling of microvasculature was examined in cortical tissue from ND and AD individuals. Representative laminin immunoreactivity (ir) for microvessels from the two categories of cases is presented in Figure 4(a) (ND, left panel; AD, right panel). Microvessels in ND showed a typical pattern of linear shape with little evidence for multiple branching from the primary vessel. A different pattern of microvasculature was manifest in AD tissue with microvessels commonly exhibiting considerable variability in length and diameter. In particular, AD tissue commonly demonstrated microvessels with short lengths (fragments) and sections with narrow and constricted diameters (see below); such properties were largely absent in ND cases. The number of microvessels/mm^2^ was determined for ND and AD cortical tissue. The results (Figure 4(b)) show that microvessel number was 72 ± 9% higher in AD, relative to ND, brain (*n* = 9 cases each for ND and AD); this difference was significant. Mean length of microvessels was significantly lower, by 18 ± 5%, in AD, relative to ND, tissue (Figure 4(c)).

#### 3.2.2. Morphological Properties of Vessels in AD Tissue

**Figure 5:**
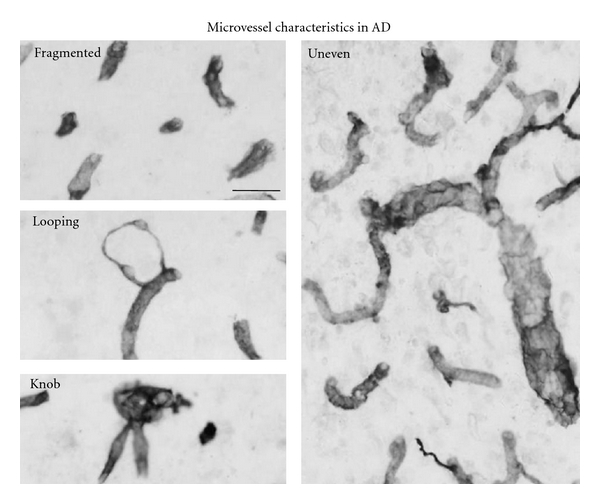
Physical and morphological properties of microvessels in AD brain tissue. The panels show microvessels with fragments, looping pattern, knob-like structure, and uneven structure. The scale bar is for 30 *μ*m.

 A pattern of altered microvessel morphology was observed between vasculature in AD and ND brain tissue. In particular, AD microvessels demonstrated a considerable variation of morphological shapes including thin fragments and nonlinear segments. Examples of specific abnormalities in AD microvessels are presented in Figure 5. Fragmented and narrow (see below) microvessels were particularly evident in AD tissue with vessels exhibiting ring-like morphology. Additionally, AD microvessels often presented as uneven diameters with areas of dilation and formation of knob-like structures. Although ND cases demonstrated microvessels with similar features to those described in AD tissue, their frequency of appearance was scarce. Previous work has documented morphological features and abnormalities in microvessels in tissue obtained from AD individuals [17–19].

#### 3.2.3. Constricted Vessels in AD Tissue

**Figure 6:**
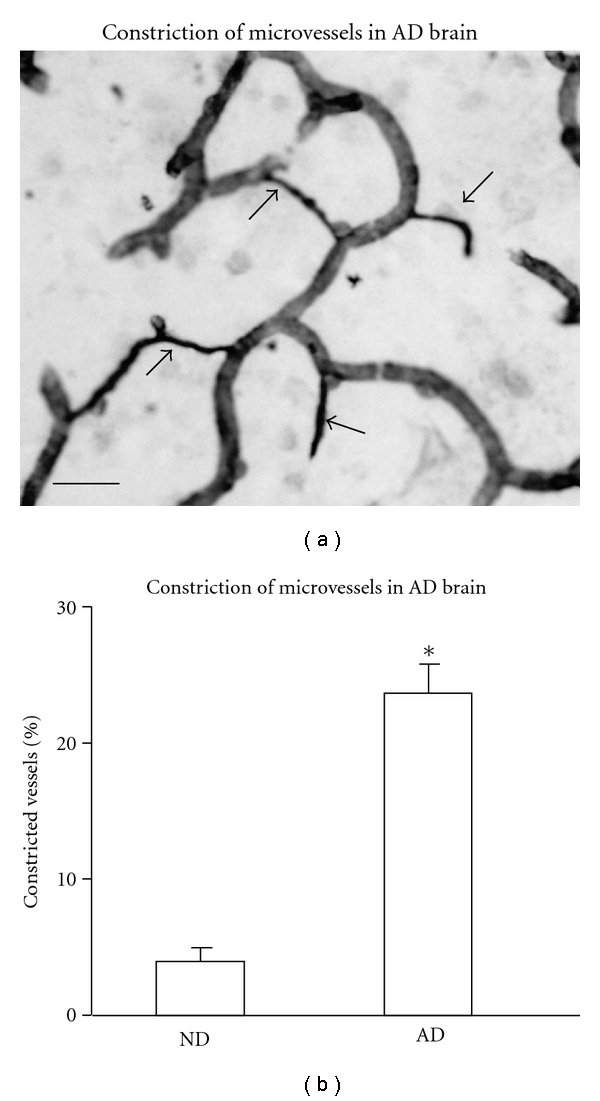
Constricted microvessels in AD brain tissue. (a) The arrows indicate constricted microvessels; scale bar is for 30 *μ*m. (b) Bar graph shows % of constricted, relative to total, microvessels for the different cases (ND, *n* = 9; AD; *n* = 9); *P ≤ 0.05 for AD versus ND.

A common finding in AD cases was the appearance of constricted microvessels in the temporal cortex as shown in the representative laminin staining (arrows, Figure 6(a)); examples of constricted microvessels were much less evident in ND cases. As for the animal model, microvessel constriction was defined as a diameter equal to, or less than, 3 *μ*m. Overall (ND, *n* = 9; AD, *n* = 9), constricted microvessels in ND cases comprised only 3.9 ± 2.2% of total vessels (Figure 6(b)). The corresponding value in AD tissue was 23.6 ± 6.7% of microvessels exhibiting constriction. Thus microvessel constriction appears as a prominent characteristic in AD brain, a distinguishing feature differentiating vasculature in diseased tissue from ND cases.

## 4. Discussion

 The primary aim of this work was to compare microvessel number, length, and morphological properties between A*β*_1−42_ and control PBS intrahippocampal injection in a rat model of AD with the same variables in human AD and ND brain tissue. The overall results show a similar pattern of microvessel perturbations in A*β* peptide-injected rat brain as present in AD brain. The changes include an increased mean number and diminished mean length of microvessels and prominent expression of microvessel abnormalities in peptide-injected rat and in AD brain tissue compared with PBS-injected and ND brain. The abnormal properties include the presence of constricted microvessels which were largely absent in controls. These results extend previous findings that the A*β*_1−42_ animal model exhibits characteristics similar to those in AD brain including an altered BBB permeability, enhanced inflammatory reactivity, and neuronal loss [12]. 

 The increased density of microvessels in A*β*_1−42_ versus PBS (rat) and AD versus ND (human) could suggest angiogenic activity in AD brain. However, a proviso is that a specific marker for angiogenesis is not well defined. Although increased expressions of laminin [20, 21] and integrin *α*v*β*3 [22, 23] have been suggested as indicators of angiogenesis they are not specific markers which differentiate between newly formed and existing capillaries. Nevertheless, a consistent enhancement in microvessel number was a common pattern of change with A*β*_1−42_ injection (versus PBS in the animal model) and in AD (versus ND) brain tissue. It is possible that angiogenic activity could be associated with increased leakiness of blood vessels. In this regard, extravasation of plasma proteins including albumin has been measured in the peptide-injected rat hippocampus [10]. 

The possibility of angiogenesis in AD brain is supported by the finding that a spectrum of proangiogenic factors is elevated in AD pathology [24]. One particular highly potent angiogenic agent is vascular endothelial growth factor (VEGF). Increased levels of VEGF are reported in microglia obtained from AD patients and in human microglia exposed to A*β*_1−42_ [11]. Furthermore, intrathecal levels of VEGF are increased in AD patients relative to controls [25]. In addition, APP23 transgenic mice show increased formation of new vessels which was inhibited using an antagonist for VEGF [26]. Taken together, accumulating evidence suggests appropriate conditions exist to support angiogenic activity in AD brain. 

Particular morphological irregularities were evident in A*β*_1−42_-injected dentate gyrus and AD brain tissue and largely absent with PBS injection and in ND brain. These abnormalities were characterized as fragmented, looping, knob-like, uneven, and constricted. Typical representations of these features are shown for A*β*_1−42_ animal treatment (Figure 2) and for human AD cases (Figure 5). Since constricted microvessels (diameters ≤ 3 *μ*m) were clearly definable compared with the other abnormal properties, it was possible to quantify their expression. Overall, about 4% of total vessels in controls were constricted compared with values of 14% in A*β*_1−42_-injected rat hippocampus and 23.6% in AD brain. It can be noted that other morphological abnormalities, such as small microvessel fragments, were evident in diseased tissue. The presence of fragments could underlie the diminished mean length of microvessels in AD brain. The altered morphology of microvessels suggests concomitant changes in cerebral blood flow in intact animals. Future studies are required to determine the effects of A*β*_1−42_ intrahippocampal injection on the hemodynamics of cerebral blood flow. Recent work using transgenic animals expressing elevated A*β*_1−42_ has reported impaired cerebral autoregulation of flow which is intact in human AD subjects [27, 28]. These studies also point out that extrapolation of animal results to describe similar processes in humans requires considerable caution. 

Overall, this work provides evidence that an animal model of A*β*_1−42_ intrahippocampal injection reproduces some microvessel perturbations which are present in AD brain. Combined with measurements on BBB dysfunction [12], the A*β*_1−42_ animal model has demonstrated efficacy in simulating prominent vasculature changes evident in diseased tissue. Future testing of other abnormal processes characteristic of AD brain such as synaptic dysfunction will help assess the overall validity of the AD animal model. In addition, prolonged exposure of brain microenvironments to A*β*_1−42_ for durations in excess of 7 d will be relevant to testing the animal model for altered vasculature and microglial-vascular interactions [16] under conditions of chronic inflammation.
